# The Highs and Lows of a Cultural Transition: A Longitudinal Analysis of Sojourner Stress and Adaptation Across 50 Countries

**DOI:** 10.1037/pspp0000046

**Published:** 2015-08

**Authors:** Kali A. Demes, Nicolas Geeraert

**Affiliations:** 1 Department of Psychology, University of Essex

**Keywords:** acculturation, stress, sojourners, adaptation, coping

## Abstract

The impact of living abroad is a topic that has intrigued researchers for almost a century, if not longer. While many acculturation phenomena have been studied over this time, the development of new research methods and statistical software in recent years means that these can be revisited and examined in a more rigorous manner. In the present study we were able to follow approximately 2,500 intercultural exchange students situated in over 50 different countries worldwide, over time both before and during their travel using online surveys. Advanced statistical analyses were employed to examine the course of sojourners stress and adjustment over time, its antecedents and consequences. By comparing a sojourner sample with a control group of nonsojourning peers we were able to highlight the uniqueness of the sojourn experience in terms of stress variability over time. Using Latent Class Growth Analysis to examine the nature of this variability revealed 5 distinct patterns of change in stress experienced by sojourners over the course of their exchange: a reverse J-curve, inverse U-curve, mild stress, minor relief, and resilience pattern. Antecedent explanatory variables for stress variability were examined using both variable-centered and person-centered analyses and evidence for the role of personality, empathy, cultural adaptation, and coping strategies was found in each case. Lastly, we examined the relationship between stress abroad with behavioral indicators of (mal)adjustment: number of family changes and early termination of the exchange program.

For decades acculturation researchers have been interested in the psychological impact on the individual, of moving to a new country (Berry, 1997; Church, 1982; Furnham & Bochner, 1986; Geeraert & Demoulin, 2013; Graves, 1967; Schwartz, Unger, Zamboanga, & Szapocznik, 2010; Ward, Bochner, & Furnham, 2001). It was originally assumed that the experience of “culture shock” was an inevitable consequence of intercultural relocation (Lysgaard, 1955). Authors such as Oberg (1960) even described culture shock as a kind of disease or condition, with symptoms such as excessive hand washing, fear of physical contact, absent-mindedness and “fits of anger.” Since this early research however, studies have shown that movement from a familiar to an unfamiliar culture does not always impact negatively. Geeraert and Demoulin (2013) for example, found that on entering the host country, the stress levels of a sample of intercultural exchange students actually decreased on average relative to pretravel levels.

The term acculturative stress is now generally preferred over culture shock when describing the impact of culture change on the individual (Berry, 1997). Rather than focusing on purely negative outcomes, acculturative stress implies a process characterized by phases of stress and adjustment (Berry, 2006). This process can be viewed within a stress and coping framework (Lazarus & Folkman, 1984) by recognizing the acculturation experience as a series of life change events (Holmes & Rahe, 1967; Spradley & Phillips, 1972). Importantly, factors identified as influential to stress and coping with other major life events, such as personality and coping strategies (Carver & Connor-Smith, 2010; Penley & Tomaka, 2002), have also been acknowledged as relevant in an acculturation context (Berry, 1997; Herman & Tetrick, 2009; Ward, Leong, & Low, 2004).

The “highs and lows” of acculturative stress, its course over time, is a central topic of exploration in the acculturation field (Berry, 1997; Ward et al., 2001). Natural partners to this topic are the questions of what can account for or explain the course of stress and adjustment over time and of what are the consequences of different acculturation experiences (Geeraert & Demoulin, 2013; Zhang & Goodson, 2011). In a multinational and longitudinal study of intercultural exchange students (of high school age) we address these issues anew by overcoming some of the limitations or boundaries of past research. Specifically, the prevalence of cross-sectional studies has limited the ability to make directional assumptions about the relationships between individual differences and cultural adjustment (e.g., Ward et al., 2004), single culture samples have restricted the generalizability of findings to other cultural groups (e.g., Wan et al., 2012), and variable-centered analyses have ignored interindividual differences in experiences within samples (e.g., Hechanova-Alampay, Beehr, Christiansen, & van Horn, 2002). The current study is the first to combine, in a single study, a longitudinal design, a multinational sample and both variable-centered and person-centered analyses. Before discussing the current research in more detail, relevant research and findings from the literature will be presented.

## Literature Overview

### Cultural Adjustment Over Time

Perhaps the most well cited theoretical model of acculturative stress and its course over time is Oberg’s (1960) culture shock model or the U-curve model of cultural adjustment (Lysgaard, 1955). Oberg (1960) described culture shock as a condition or state with both physical and psychological symptoms. Importantly, he argued that not all individuals may experience these symptoms with the same intensity or at the same time. However, the model does specify a number of stages through which every acculturating individual will travel. The first is one of euphoria and fascination with the new culture, labeled the “honeymoon.” If the person remains in the new culture long enough, a second “crisis” stage begins, characterized by hostile attitudes toward the host culture. However, as the individual gradually learns about and opens up to the culture, a phase of recovery occurs, ending finally in full adjustment and acceptance of the culture. While somewhat anecdotal in its basis, this model does hold intuitive appeal and has been tested frequently since its specification (Black & Mendenhall, 1991; Gullahorn & Gullahorn, 1963; Hechanova-Alampay, Beehr, Christiansen, and van Horn, 2002; Ward, Okura, Kennedy, & Kojima, 1998). Overall, however, support for this U-curve or stage pattern of cultural adjustment is mixed.

In a sample of international students at an American university, Hechanova-Alampay et al. (2002) did observe an inverse U-curve pattern of strain over three time points from the start of a semester, to 3 and 6 months later. Ward et al. (1998), in line with a stress and coping prediction (that initial entry to a new country will involve the most life changes and thus the most stress), found support for an alternative pattern of cultural adjustment, a reverse J-curve. Specifically, in a sample of Japanese students in New Zealand social difficulty and depression was found to be at its highest 24 hours after entry, dropping steeply at 4 months and leveling over the remaining waves. Other patterns have also been reported, such as drops in sojourners stress on arrival to the host country relative to baseline levels (Geeraert & Demoulin, 2013).

Given the variety of adjustment patterns that have been observed across different samples of intercultural travelers, one could ask whether there really is a single “one size fits all” description of how cultural adjustment proceeds over time. We argue that this may not be the case and that a person-centered approach to such longitudinal data may be a more appropriate way to assess this. While variable-centered approaches (e.g., factor analysis, multilevel modeling) describe how variables relate to one another, person-centered approaches (e.g., cluster analysis, latent class analysis) describe how individuals relate to one another (Jung & Wickrama, 2008; Muthén & Muthén, 2000). Specifically, in our study we attempt to identify a number of subgroups of individuals experiencing different patterns of cultural stress and adjustment over time.

This type of approach was employed by Wang, Heppner, Fu, Zhao, and Chuang (2012), in a study of Chinese international students in the U.S., using growth mixture modeling as a means to identify distinct trajectories of psychological distress measured over four time points. They identified four subgroups of individuals: a “well-adjusted” group reporting consistently low distress levels, a “culture-shocked” group experiencing a peak in distress during their first semester, a “relieved” group who reported a drop in stress from prearrival to the first semester and a final “consistently distressed” group. Interestingly, Wang et al. (2012) found that individuals belonging to these different subgroups could be distinguished along variables measured pre- and postarrival to the U.S., including self-esteem, perfectionism, and coping strategy use.

### Antecedents of Cultural Adjustment

A natural progression from the examination of cultural stress and adjustment over time is to explore what variables moderate that adjustment; that is variables that appear to facilitate or hinder the acculturation process. In line with the stress and coping framework, previous research has examined the role of variables such as expectations (Martin, Bradford, & Rohrlich, 1995; Demes & Geeraert, 2015), personality (Swagler & Jome, 2005; Ward, Leong, & Low, 2004), social support (Cemalcilar, Falbo, & Stapleton, 2005; Geeraert & Demoulin, 2013), and coping strategies (Wang et al., 2012; Ward & Kennedy, 2001). In this article we focus on the role of personality and coping strategies (including social support) in explaining different patterns of stress during a cross-cultural transition. We will also examine the function of empathy, an interpersonal tendency related to personality, in the adjustment of our sojourners.

#### Personality

In Berry’s (1997) elaborated stress and coping model of acculturation, personality is specified as an important moderating factor that is present preacculturation. Locus of control (Ward & Kennedy, 1992) and self-efficacy (Harrison, Chadwick & Scales, 1996) for example, are among several traits that have been studied in relation to the success of a cultural transition. One of the most famous and popular conceptualizations of personality, however, is the Five Factor Model, also known as the Big Five (Costa & McCrae, 1992; Piedmont, 1998). This describes five central personality traits: openness, conscientiousness, extraversion, agreeableness, and neuroticism. Similar to this but more recent is the HEXACO personality model describing six major personality dimensions (Ashton & Lee, 2009). Five of the six factors of the HEXACO broadly overlap with the Big Five concepts, but a sixth novel factor labeled honesty-humility is also specified (Ashton & Lee, 2001). This six factor structure has been shown to replicate well across cultures and honesty-humility is believed to be a valuable addition to the model from a cross-cultural perspective (Ashton & Lee, 2007).

To date some research has examined the relationship between the Big Five personality traits and the cultural adjustment of sojourners. For example, Swagler and Jome (2005), in a sample of North American sojourners in Taiwan, reported a negative association between psychological adjustment and neuroticism but a positive association with agreeableness and conscientiousness. In addition they found that extraversion was positively related to sociocultural adjustment. Ward, Leong, and Low (2004) also found that neuroticism was negatively and extraversion positively related to the cultural adaptation of sojourners in Australia and Singapore and reported evidence linking agreeableness and conscientiousness to sojourners well-being.

While these findings support the inclusion of personality in acculturation models, these are based almost exclusively on cross-sectional research (Berry, 1997; Swagler & Jome, 2005; van der Zee & van Oudenhoven, 2013; Ward et al., 2004). Consequently, these studies have not been able to attest to the durability or stability of these effects over time. The present study, therefore, provides a crucial progression from previous research by examining the influence of personality on *change* in adjustment over time using longitudinal research methods. Additionally, utilizing the HEXACO personality inventory (Ashton & Lee, 2009) as opposed to the Big Five Model, allows us to examine whether a sixth personality factor, honesty-humility, has a role to play in cultural stress and adjustment (Ashton & Lee, 2007). Previous research suggests that low levels of honesty-humility are related to increased levels of narcissism, Machiavellianism and psychopathy (Lee & Ashton, 2005), traits that play an important role in interpersonal settings (Holden, Zeigler-Hill, Pham, & Shackelford, 2014). Considering the role of honesty-humility in sojourner adjustment may be particularly relevant as a key feature of an intercultural exchange is to form brand new relationships in the host country.

#### Empathy

While not explicitly specified in Berry’s (1997) stress and coping model of acculturation, empathy has associations with emotion regulation which is relevant from a stress and coping perspective (Matsumoto, Hirayama, & LeRoux, 2006). We are interested to see how sojourners levels of empathy may relate to their stress and adjustment patterns over the exchange and to do this draw upon a multidimensional conceptualization of empathy (Davis, 1983). We are particularly interested in the perspective taking and empathetic concern dimensions due to their associations with positive interpersonal functioning (Davis, 1983; Galinsky, Ku, & Wang, 2005; Håkansson & Montgomery, 2003). Perspective taking has been described as the cognitive ability to take on the perspective of another person, while empathetic concern is a more emotional tendency that involves feeling sympathy and concern for others (Davis, 1983).

The relevance of these traits or tendencies to intercultural relocation is clear. Part of the adjustment process to living abroad involves understanding people who come from a different cultural background who may think about and perceive the world in a very different way but at the same time also experience the same feelings, concerns, or joys as anyone else. Indeed, Ward and Kennedy’s (1999) Socio-Cultural Adaptation Scale includes a cultural empathy and relatedness factor, as does the Multicultural Personality Questionnaire of van der Zee and van Oudenhoven (2000), highlighting its relevance to cultural adjustment. In a study of students at two international business schools, Van Oudenhoven and van der Zee (2002) found that scoring higher on the cultural empathy factor of the Multicultural Personality Questionnaire was related to reporting better physical and mental health, higher subjective well-being and higher perceived peer support.

While cultural empathy is described as functional for cultural adjustment, the empathetic concern factor of the Interpersonal Reactivity Index (Davis, 1980) has been associated with emotional vulnerability (Davis, 1983), a trait which may actually hinder cultural adjustment. For example, Matsumoto, Hirayama, and LeRoux (2006) highlight the importance of emotion regulation in sojourner adjustment, which is a facet in their Intercultural Adjustment Potential Scale (Matsumoto et al., 2001). Therefore, high levels of empathetic concern may be detrimental in this context. Perspective taking as a discrete facet of empathy has received little direct attention in the acculturation literature. However, more general research on perspective taking suggests that it is positively related to social competence (Davis, 1983) and the development of social bonds (Galinsky et al., 2005). Given that the perspective taking and empathetic concern dimensions of the Interpersonal Reactivity Index (Davis, 1980) have not yet been examined in the context of sojourner adjustment, our research may shed more light on the role of these discrete facets of empathy.

#### Coping strategies

When a situation is appraised as problematic, that is as causing stress, individuals employ coping strategies with the aim to ameliorate the stress (Lazarus & Folkman, 1984). While some coping strategies might have short-term benefits, not all are effective at reducing stress in the long-term (Carver, Scheier & Weintraub, 1989). A number of different models of coping styles have been specified in the literature, distinguishing between functional and dysfunctional types. Problem-focused and emotion-focused coping distinguish between strategies that address and target the problem “head on” and strategies that aim to address the symptoms (i.e., stress) caused by the problem, respectively (Lazarus & Folkman, 1984). Other distinctions have been made between approach and avoidance coping (Nes & Segerstrom, 2006), active versus passive (Diaz-Guerrero, 1979), and primary and secondary control coping strategies (Skinner, Edge, Altman, & Sherwood, 2003). There is a great deal of overlap, however, in how each of these contrasting coping styles is defined. Specifically, the remit of problem-focused coping is similar to that of approach, active, and primary control coping, while there is overlap between emotion-focused and avoidance coping. Passive coping and secondary control coping are similar to one another in that they both aim to alter the self in some way in order to better adjust to the situation (Skinner et al., 2003); this has been equated with the assimilation approach to cultural adjustment (Berry, 1997). Other examples of coping strategies that do not directly fall within one of the above styles include turning to religion, using humor, self-blaming, and seeking social support (Carver et al., 1989; Ward & Kennedy, 2001).

Typically, problem-focused or approach strategies are associated with positive adjustment outcomes and avoidance or emotion-focused strategies with negative outcomes. For example, Herman and Tetrick (2009) studied the repatriation adjustment of North American and Australian employees who had worked in Japan. In a cross-sectional investigation, they found that problem-focused coping was related to better adjustment on return, while emotion-focused coping was related to poorer adjustment. Similarly, in a cross-sectional study of British citizens in Singapore, Ward and Kennedy (2001) found that approach and avoidance strategies respectively, were negatively and positively related to depression (psychological adjustment). They also found that the use of humor was related to positive adjustment but found no effect of social support.

Overall there is little research in the field that has examined the association between the use of different coping strategies and cultural adjustment in student sojourner samples, (i.e., international university students or exchange students) and even fewer that have employed longitudinal research methods. An exception to this on both counts is research by Wang et al. (2012), who studied the relationship between different coping styles and psychological distress trajectories in Asian students in the U.S. They found that the use of acceptance, reframing, and striving strategies as well as family support were related to better adjustment. They found no effect, however, of the use of avoidance, religion, or emotional outlets on adjustment. A major strength of their research is its longitudinal design, which allowed for assumptions to be made about the relationship between coping and adjustment over time. Still, their research was also limited in that it studied a single cultural group within a single cultural setting. Therefore, the observed effects may be unique to this particular combination of sending and hosting cultures and generalization to other groups may be problematic. This is one of the limitations that the present research overcomes.

### Consequences of Cultural Maladjustment

A further area of study in this field concerns the behavioral consequences of adjustment or maladjustment. Specifically, one of the main signs of failure of an intercultural exchange is early termination of the program. However, very little research has monitored and investigated early return in student samples. Most research on this issue in fact focuses on international assignees or expatriate samples (e.g., Black & Gregerson, 1990; Caligiuri, 2000). Failed international assignments can be financially costly for the sending organization (see Mendenhall, Dunbar, & Oddou, 1987) and can jeopardize employees future career prospects (Bolino, 2007). For exchange students, returning home early may be equally detrimental. However, the cost is likely to be more personal and psychological in nature than financial or career related. Nevertheless, while the precursors to or warning signs of early return have received attention in research on expatriates (Caligiuri, 2000; Hechanova, Beehr, & Christiansen, 2003), this has not been the case for other sojourning populations such as exchange students.

Certainly, research has explored and identified predictors of good or poor cultural adjustment in students (Zhang & Goodson, 2011), but the link between adjustment and early return has rarely been examined directly. One exception to this is a longitudinal study of Belgian exchange students by Geeraert and Demoulin (2013). They found a significant and positive association between acculturative stress and early return, such that higher stress in the early phases of the exchange predicted increased likelihood of subsequent early return. A particular strength of their study was that actual early return was recorded rather than intention or desire to return which is most typical in expatriate research (e.g., Black & Gregerson, 1990; Caligiuri, 2000). Still, while this finding is important, the researchers acknowledge that its implications are limited, as the sample size of the early return group was very small (*n* = 10). Consequently, the current study provides the ideal conditions to study this further as the size of our sample is larger.

In addition to early return, in exchange student samples that live with a host family while abroad, another behavioral indicator of (mal)adjustment is changing host families. To our knowledge, however, there is no published research that has examined this variable and its association with stress. In this study we examine the relationship between stress and coping and the number of family changes experienced by participants.

## Current Research

In this article we present findings from a longitudinal study of approximately 2,500 intercultural exchange students of multiple nationalities who were situated in over 50 different countries for a year abroad. Taking advantage of the longitudinal and multinational nature of this study’s design and of appropriate advanced statistical techniques, we investigate a number of research questions that are central to the acculturation field. First, we will examine the variability in stress over time in a sojourning and nonsojourning group of participants. The nature of any variability found will be decomposed using a person-centered approach to assess interindividual differences in temporal stress trajectories. Specifically, steering away from trying to identify a single standard course of adjustment over time (e.g., Oberg, 1960; Hechanova-Alampay et al., 2002), we will investigate whether multiple discrete trajectories of change in stress can be identified within our sample. Given that our sample includes individuals traveling from and traveling to multiple different countries means that we can generalize our findings more broadly than has been possible in previous research (e.g., Wang et al., 2012).

Second, we will look at whether personality, empathy, cultural adaptation, and coping strategies can explain variations in stress over time and account for the likelihood of experiencing different patterns of stress. Importantly, and as acknowledged by other researchers, personality and coping strategy use as antecedents of cultural adjustment have been relatively understudied, especially in a sojourner population and in longitudinal research studies (Ward et al., 2001; Ward & Kennedy, 2001).

Third and finally, we will assess whether the different stress and adjustment patterns identified relate to two behavioral measures concerned with cultural (mal)adjustment: early return and host family changes. The examination of behavioral indicators of adjustment is rare in the literature making these measures an exciting addition to the present research. In the following section the research method and design will be described. The results will then be presented in three discrete sections concerning (a) stress over time, (b) their antecedents, and (c) consequences; each section includes a short introduction with predictions, a Results section, and a Discussion section.

## Method

### Participants

A sample of 2,480 teenagers (*M*_*age*_ = 17.0 years, *SD* = 1.4 years, 70% female) who participated in an intercultural exchange program were followed by the researchers over an 18-month period, from 2 months before, through to 6 months postexchange. These participants were registered with the organization AFS Intercultural Programs,^1^ a nonprofit, volunteer-based organization that offers international exchange programs in more than 50 different countries. Typically, these students are placed with a host family for the duration of their 8–10 month stay abroad and during this time they enroll at a local high school. The participants to this project were traveling from one of 46 different “home” countries to one of 51 different “host” destinations. Table 1 provides a breakdown of sample size per sending (home) and hosting countries. In addition to the sojourner participants, data was collected in parallel from a nonsojourning control group, who were friends nominated to take part in the study by the sojourner participants (*N* = 578, *M*_*age*_ = 17.7 years, *SD* = 2.1 years, 74% female).[Table-anchor tbl1]

### Design and Procedure

Data was collected from a large-scale longitudinal study with a total of nine timewaves of measurement, and both a sojourner and control group. For the purpose of the current research we focus only on data collected at the pretravel and exchange data waves (Timewaves 1 to 6). Timewaves 1 and 2 were recorded approximately 3 months and 1 month prior to participants traveling abroad (pretravel), respectively. Timewaves 3, 4, 5, and 6 were measured during the sojourn, approximately 2 weeks (t3), 2.5 months (t4), 5 months (t5), and 8.5 months (t6) after arrival to the host country. At each timewave, participants were invited by e-mail to visit the project website, log in, and complete an online survey. The timeline for the control group’s survey assignments was matched with that of the sojourner who nominated them.

To encourage ongoing participation in the study, a number of incentives were offered over the course of the project, these were bimonthly prize draws, an end of project “grand” prize draw and personalized feedback. If participants missed a survey, they were still invited to complete the subsequent one. Overall, participant retention was respectable; of the 2,480 sojourners who started the study, 1,141 completed the final survey, and of those, 826 had completed all nine surveys over the duration of the project. Overall, the average number of surveys that participants in the sojourning group completed was 5.92 (*SD* = 3.00) and in the nonsojourning control group 5.00 (*SD* = 2.91).

### Materials

All survey materials were translated in to 10 different languages from English, covering the most commonly spoken languages within the AFS Network. Materials were translated in to Chinese (simplified and traditional script), French, German, Italian, Japanese, Portuguese, Spanish, Thai, and Turkish using the standard forward-backward translation procedure (Brislin, 1980).

Over 20 different concepts were recorded through the online surveys but here we only concentrate on those measures relevant to the present research questions. These measures, all in the format of 7-point Likert-type scales, include a measure of stress, cultural adaptation (sociocultural and psychological adaptation), personality, interpersonal reactivity (perspective taking and empathetic concern), and coping. Higher scores on each scale indicate higher levels on that construct, such as higher stress, greater adaptation, and so forth. Additionally, data for two behavioral measures, early return and family change, were acquired directly from the exchange organization and matched with participants survey responses.

#### Stress

At each timewave, a brief version of the perceived stress scale (Cohen, Kamarck, & Mermelstein, 1983) was administered. Participants were asked “*In the last 2 weeks how often have you felt* . . .” which was followed by four items such as “*you were unable to control the important things in your life*.” Participants responded using a 7 point scale (1 = never, 7 = always) and reliability at each wave was good (all α’s > .70).

#### Cultural adaptation

Two scales were used to assess sojourners’ sociocultural and psychological adaptation (Ward & Kennedy, 1996). Adaptation was recorded twice during the sojourn, once at the start (t3) and once half-way through the sojourn (t5). Adaptation was not measured at t4 or t6, but for the purpose of the analyses, adaptation scores for these timewaves were assigned from the previous timewave (t3 and t5, respectively).

The Brief Sociocultural Adaptation Scale (Demes & Geeraert, 2014) was used to assess ease of adaption to social and cultural elements of the host country. Put differently, this measure assesses the behavioral and practical aspects of sojourners’ adaptation. Participants were asked “*How easy or difficult do you think it is for you to adapt to* . . .” which was followed by an item such as “*social norms (how to behave in public, style of clothes, what people think is funny*).” Sojourners responded using a 7-point scale (1 = very difficult, 7 = very easy). A total of 12 items assessed adaptation to such topics as climate, language, making friends, the food, and pace of life (α_*t3*_ = .85, α_*t5*_ = .82).

The Brief Psychological Adaptation Scale (Demes & Geeraert, 2014) was used to assess the emotional and psychological aspects specific to a cultural relocation, rather than psychological adaptation in the broader sense. Participants were asked “*In the last 2 weeks how often have you felt* . . .” followed by items such as “*excited about being in* [name of the host country]” and “*homesick when you think of* [name of the home country].” Across eight items the scale assesses the frequency of experiencing happiness, excitement, but also homesickness or feeling out of place (α_*t3*_ = .83, α_*t5*_ = .83).

#### Personality

A six factor personality structure was measured prior to the sojourn (t1) using the HEXACO inventory (Ashton & Lee, 2009). Across 60 items participants had to indicate their agreement with items such as “*I would never accept a bribe, even if it were very large*” (honesty-humility), “*I feel like crying when I see other people crying*” (emotionality), “*The first thing that I always do in a new place is to make friends*” (extraversion), “*Most people tend to get angry more quickly than I do*” (agreeableness), “*I often push myself very hard when trying to achieve a goal*” (conscientiousness), and “*People have often told me that I have a good imagination*” (openness to experience). All six factors had good reliability (all α‘s > .70, except α_*agreeableness*_ = .67).

#### Interpersonal reactivity

Perspective taking and empathetic concern were measured prior to the sojourn (t1) using the Interpersonal Reactivity Index (Davis, 1980). Participants had to indicate their agreement with items such as “*I try to look at everybody’s side of a disagreement before I make a decision*” (perspective taking, α = .71) and “*I am often quite touched by things that I see happen*” (empathetic concern, α = .72).

#### Coping strategies

The brief COPE scale (Carver, 1997) was implemented to assess the reported frequency with which participants used different coping strategies during the exchange (t4). Specifically, participants were asked to “*Think about any difficult times you have experienced since you arrived in* [name of the host country]. *How often do you have the following reactions?*” A series of items assessing the different coping strategies of the COPE scale were then presented, with two items for each of the following strategies: acceptance, active coping, disengagement, denial, distraction, humor, planning, reframing, religion, self-blame, substance abuse, emotional support, instrumental support, and venting. In the context of acculturation a distinction is often made between support received from host nationals versus home nationals (Geeraert, Demoulin, & Demes, 2014). In light of the Internet and social media and the ease of communicating across large geographical distances, in this study we took a related but alternative approach and assessed support received from people locally in the host country (*close support*) separately from support received remotely from people in the home country (*distant support*). This required altering the support items to reflect this. For example, one of the two items for emotional support was altered from “*I get emotional support from others,*” into “*I get emotional support from people I’ve met in* [name of the host country]” for close support and into “*I get emotional support from people back in* [name of the home country]” for distant support. For the instrumental items, rather than “*I try to get help and advice from people,*” this particular item read “*I try to get help and advice from people I’ve met in* [name of the host country]” for close support and “*I try to get help and advice from people back in* [name of the home country]” for distant support. Thus, both emotional and instrumental support items were measured for both geographically close and distant support, resulting in a total of 16 coping strategies being assessed overall.

Preliminary inspection of the coping strategies’ internal consistency and external validity indicated some issues with certain strategies. Specifically, internal consistency was particularly low for venting (α = .22) and distraction (α = .48). Also, bivariate correlations between coping strategies and stress, as an indicator of validity, were particularly low for religion (*r* = .05), humor (*r* = −.09) and distraction (*r* = .09). We decided to remove these four coping strategies, resulting in 12 remaining strategies with acceptable reliability (all α’s > .60, except for α_*acceptance*_ = .54) and external validity (all *r*’s > +/−.15). Next, in the interest of further data reduction we examined the factor structure of the scales to see whether these strategies might be collated at a higher level. Based on methods employed in previous research using the COPE scale (e.g., Carver et al., 1989; Deisinger, Cassisi, & Whitaker, 1996; Ward & Kennedy, 2001), we expected the active coping and planning items to be explained by a single factor, similarly so for disengagement and denial, acceptance and positive reframing, and then the emotional and instrumental support items, each for close and distant support. We did not have any predictions for the substance abuse and self-blame strategies. The predicted factor structure emerged both in a first order factor analysis (on the 24 items) and a second-order factor analysis (on the 12 strategies).^2^ Thus, we combined active coping and planning into an approach strategy (α = .73), denial and disengagement into an avoidance strategy (α = .72), positive reframing and acceptance into an acceptance strategy (α = .65), emotional and instrumental support in the host country into a close support strategy (α = .92), and emotional and instrumental support in the home country into a distant support strategy (α = .93). Self-blame (*r* = .58) and substance abuse (*r* = .81) were upheld as separate strategies. These seven higher order factors provide a more parsimonious conceptualization of coping strategy use than the original 16 and will be utilized in the analyses.

#### Behavioral measures

Data regarding early return from the sojourn and host family changes was acquired directly from the exchange program organizers. Early return is a dichotomous variable coded as 1 or 0, 1 meaning that a student did return home early and 0 meaning that they did not. Out of the 2,480 participants, 111 returned home early, a 4% rate overall. Family change is a continuous variable, from 1 upward that serves as a count of how many host families students resided with. Although the majority of sojourners stayed with a single family throughout their exchange (*N* = 1,760, 71.0%), others changed families once (*N* = 499, 20.1%), twice (*N* = 176, 7.1%), three times (*N* = 33, 1.3%), or more (*N* = 12, .5%). For the whole sample of sojourners the average number of host families was 1.4 (*SD* = .74).

### Statistical Considerations

Of the 2,480 sojourners in this sample, 2,015 were eligible for the following analyses and of the 578 controls, 447 were eligible. The remaining participants were excluded due to having completed none of the surveys during the sojourn phase. To assess the nature of this missing data we examined whether there was an association between pretravel measures and attrition. Attrition from the study was more likely for male participants (*p* < .001), and for those with lower scores on honesty-humility or conscientiousness (*p*’s < .001). Importantly however, attrition was not associated with stress (*p* > .10). Thus, although the *missing completely at random* (MCAR) assumption was not met, we can assume that missing data due to attrition was *missing at random* (MAR; but see Hox, 2010). Although some participants did not complete every survey, data from their completed surveys was still included in the analyses. The advantage of many advanced statistical techniques is their ability to handle missing data, which is crucial for longitudinal research. Having met the conditions for MAR this should satisfy the preconditions for handling missing data in the following analyses (such as FIML).

A further consideration for this study is the level at which effects are deemed to be statistically significant. Due to the large sample size (*N* > 2,000), there is an increased chance of finding significant effects at the conventional level of *p* < .05, where no real effect actually exists (i.e., incorrect rejection of the null hypothesis or Type I error). Therefore, in all of the following analyses we employ the stricter significance threshold of *p* < .005. However, where planned contrasts are conducted and are protected by a significant omnibus effect at the strict threshold (*p* < .005), the conventional significance level (*p* < .05) will be applied.

## Stress Over Time

A significant area of research in the acculturation field has been dedicated to examining the stress, well-being, and adjustment of sojourning groups over the duration of an intercultural relocation. While some research has attempted to compare sojourning with nonsojourning populations along these variables, as a means to assess the “uniqueness” of the sojourning experience, the logistics of longitudinal research that captures both samples mean that such studies are still rare (e.g., Geeraert & Demoulin, 2013). The design of the present study however allows us to compare change in stress levels over time in both a sojourning and a nonsojourning control group. We expect to find more variability in the sojourning group with regards to stress over time than in the control group.

**Figure 1 fig1:**
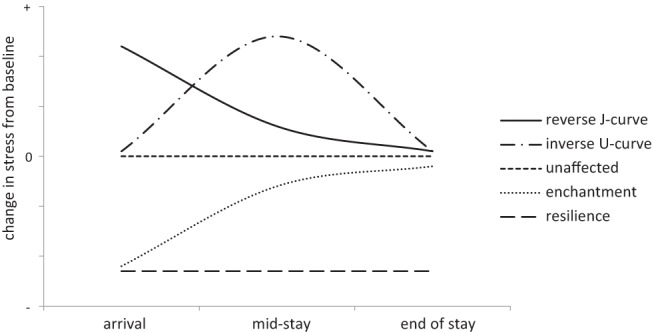
Hypothesized stress trajectories showing five different predicted patterns of change in stress over the exchange relative to baseline stress (anchored at 0 on the *y*-axis). Note: Above 0 on the *y*-axis indicates an increase in stress relative to baseline and below 0 on the *y*-axis indicates a decrease in stress relative to baseline.

The particular pattern or nature of this variation in stress and adjustment over time has also been a central theme in acculturation research. However, past attempts to identify a single temporal pattern of sojourner stress and adjustment have proved inconsistent across studies (Geeraert & Demoulin, 2013; Hechanova-Alampay et al., 2002; Ward et al., 1998). Rather than seeking a “one size fits all” solution to this question, we employ a person-centered approach to examine interindividual differences within our sojourner sample in relation to change in stress over time. Using this approach we anticipate finding up to five distinct temporal stress patterns as based on research and findings from earlier studies (see Figure 1).[Fig-anchor fig1]

Relative to pretravel stress, some participants are expected to experience an increase in stress immediately upon arrival to the host country, followed by a steady decrease over time; this is a typical “J-curve” of adaptation or reverse “J-curve” of stress (Ward et al., 1998). Other participants are anticipated to demonstrate a more gradual increase in stress over time that peaks around midstay and drops later on, in line with “U-curve of adaptation” or inverse “U-curve” of stress (Hechanova-Alampay et al., 2002). Additionally, it is predicted that some participants will actually demonstrate a decrease in stress on arrival to the host country that remains stable over time: a “resilience” pattern (Geeraert & Demoulin, 2013). Further, drawing upon the work of Diener and colleagues on subjective well-being and the return to “dispositional” levels following significant life events (Diener, Suh, Lucas, & Smith, 1999), an “enchantment” response is anticipated from some participants where stress levels initially decrease on arrival but then return to baseline over time. Finally, we also expect that some participants will show very little stress response to the exchange, that is, an “unaffected” or stable pattern of stress.

Our design allows us the opportunity to compare sojourner stress trajectories with those of a nonsojourning control group. Unlike for sojourners however, we have no theoretical basis on which to predict particular stress trajectories for the controls. Also, we do not have any information regarding possible life events that would have affected individuals with the control sample. We do expect that there will be some interindividual differences within the control group over time but that these patterns will be more uniform overall when compared with the sojourner group.

### Results

#### Sojourner stress trajectories

Change in sojourners stress over the course of the exchange was examined using a person-centered approach, moving away from attempts to identify a single growth curve to chart sojourner stress over time. Specifically, we explored whether multiple growth curves can be identified among sojourners using latent class growth analysis (LCGA; Jung & Wickrama, 2008) conducted in MPlus 7.11 (Muthén & Muthén, 2012). As in standard latent growth curve analysis, LCGA models patterns of change in a particular variable over time. However, rather than estimating a single latent intercept and slope to describe the full sample, LCGA allows specifying multiple latent intercepts and slopes that describe different patterns of change across meaningful subpopulations or classes within the larger sample.^3^

The number of subsamples or classes that are modeled in LCGA is user-defined. Practically, the user builds up the model by specifying the minimum number of classes in the first instance, and then increases by one class at a time in subsequent models. Comparing model fit statistics (e.g., BIC) and likelihood ratio tests between models with *k* - 1 class (i.e., models with one less class) guides identification of the most suitable or best fitting number of classes for the data. In addition, it is crucial that model selection is also guided by theory and prior predictions about the number and nature of the classes expected. It is important therefore to inspect the class growth plots specified by the models. In sum, model selection is guided by a combined evaluation of the statistical output, prior predictions and interpretability of classes.

##### Specifying the LCGA model

Stress measured at four time points during the exchange (t3, t4, t5, and t6) was modeled using LCGA. Crucially, we were specifically interested in identifying patterns of change in stress upon entering the host country relative to pretravel levels, rather than absolute levels of stress. Absolute levels of stress are likely to vary according to individual and cultural level differences that we are not interested in capturing here, but that will be addressed in later analyses. Therefore, for the purpose of this analysis we centered stress recorded during the exchange (t3 to t6) on baseline stress (an average of the two pretravel records of stress, t1 and t2).^4^ This means that each individuals stress score at the four time points was a measure of deviation from baseline, with positive values representing increases in stress, and negative values representing decreases in stress from pretravel.

As described above we expect to find up to five distinct trajectories of stress within the data (see Figure 1). Consequently, a series of models were conducted that specified two through to six classes. We constructed a six-class model to see whether there is a further meaningful class that we had not anticipated. In addition, given that four time points were analyzed and that nonlinear trajectories were expected (i.e., U-curve), linear models in each case were compared with a model including a quadratic growth factor. This resulted in a total of 10 models, each specifying the full-information maximum likelihood algorithm for handling missing data.

##### Model comparisons

Selection of the final model was guided by comparing the recommended fit statistics between alternative models (see Table 2) including BIC, sample size adjusted BIC (aBIC), entropy, and latent class fit probabilities (Jung & Wickrama, 2008).^5^ In addition to, and complimentary to this, the class growth plots were inspected for each model and judged according to their interpretability in light of the hypothesized trajectories. We preferred the five-class quadratic model over all others, for a number of reasons. First, this model had a smaller BIC and aBIC than equivalent models with fewer classes and the BLRT suggested that the five-class model was a significant improvement over the four-class model. Second, the latent class fit probabilities were .75 or above for all of the five classes, indicating a good fit for the data within each class. Third, inspection of the growth plots for the five-class quadratic model revealed that the classes were interpretable in light of the hypothesized trajectories and prior research. Although there were slight improvements in the fit statistics for the six-class quadratic model, the sample size for the smallest class was very low (*n* = 45), and importantly, visual inspection of the six-class solution was not clearly interpretable in light of previous theory and research.[Table-anchor tbl2]

##### The final model

**Figure 2 fig2:**
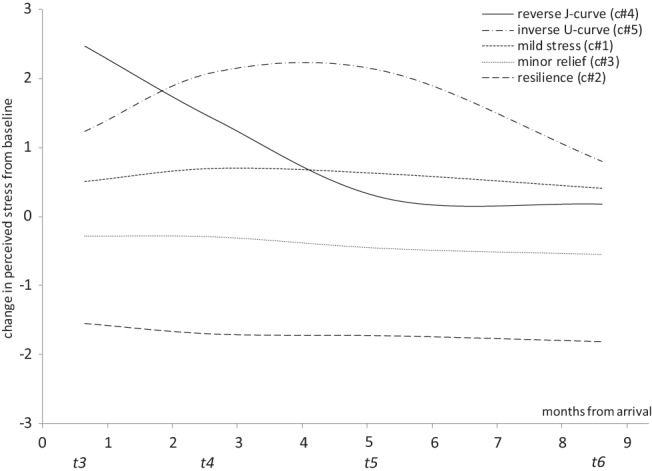
Five class representation of change in perceived stress over time for sojourners. Change in stress is relative to pretravel baseline.

The five growth trajectories from the five-class quadratic model were plotted and are presented in Figure 2. As participants stress levels at each time point were centered on baseline stress, 0 on the *y*-axis represents the baseline, positive values represent increases in stress from baseline and negative values represent decreases in stress from baseline. Each class was assigned a label describing the nature of the pattern of change in stress, as follows. The analysis revealed the anticipated reverse J-curve pattern of change in stress for 64 individuals (3.2%). The analysis also yielded the hypothesized inverse U-curve pattern of change in stress for 98 individuals (4.9%). Although a single “stable” class was not identified by the model, the two largest classes showed only small deviations from baseline in each direction and thus represent two groups with relatively stable levels of stress over the course of the study. A “mild stress” class included 819 individuals (40.6%) who demonstrated a small but relatively stable elevation in their stress during the exchange. Additionally, a “minor relief” class represents 951 individuals (47.2%) who reported a small and stable drop in stress from baseline during their exchange. Finally, the predicted “resilience” pattern was observed which depicted a marked and stable decrease in stress from baseline for 83 individuals (4.1%). The hypothesized “enchantment” group, that is, a group of participants’ who first dropped in stress and then returned to baseline, was not observed in the data. Overall, however, the five classes derived from this model are consistent with the initial predictions.[Fig-anchor fig2]

#### Control group stress trajectories

**Figure 3 fig3:**
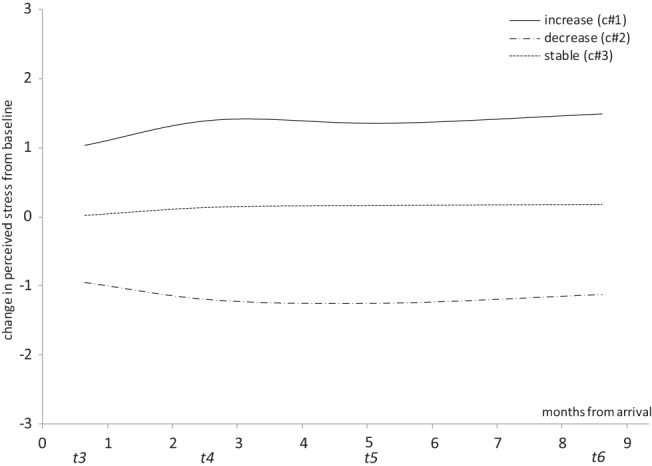
Three class representation of change in perceived stress over time for controls. Change in stress is relative to pretravel baseline.

In order to assess whether the sojourner group experienced greater interindividual variation in stress trajectories over time than the nonsojourning control group we replicated the LCGA analysis described above on the control group data. As anticipated, fewer meaningful stress trajectory classes were identified in the control group than in the sojourner group. Specifically, the statistical output of the LCGA analysis suggests that a linear three-class solution is most appropriate (see Table 3). The three-class solution was preferred as the final model given its smaller BIC and aBIC indices and greater entropy value when compared with the equivalent two-class solution. Further, the LMR-LRT and BLRT both indicate a significant improvement in the three-class model from the two-class. While the fit statistics suggest a very minor improvement for the four-class solution over the three, this is rejected due to the very small sample size of the fourth class (*n* = 7). Finally, the linear solution is selected over the quadratic as there is very little change or improvement in the fit statistics in the latter. Examining the growth plots for the three-class linear solution (see Figure 3) highlights that the majority of control participants (*n* = 312) did not demonstrate any shift in stress from baseline levels over the subsequent measurement waves. The two remaining classes represent smaller samples of individuals who reported a slight linear increase (*n* = 88) or decrease (*n* = 47) in stress over time relative to baseline levels. Importantly, these changes in stress had a smaller range than for the sojourners.[Table-anchor tbl3][Fig-anchor fig3]

### Discussion

The pattern of stress experienced by participants over the course of this study was explored using LCGA to model multiple growth curves depicting varying patterns of change in stress over time, relative to baseline stress in both the sojourning and nonsojourning control group. This analysis revealed five statistically meaningful and theoretically interpretable growth trajectories of stress within our sojourner sample, supporting our argument that specifying a single standard temporal pattern may ignore important variability within sojourning groups (Geeraert & Demoulin, 2013; Hechanova-Alampay et al., 2002; Ward et al., 1998). Specifically, while some participants showed marked increases in stress on arrival to the host country (i.e., the reverse J-curve and inverse U-curve patterns), others clearly decreased in stress (i.e., the resilience pattern). We did not, however, find the hypothesized “enchantment” group, as would be anticipated if participants experienced a “honeymoon” phase of low stress on entry to the host culture. While this effect has been discussed theoretically and anecdotally in previous literature (Oberg, 1960) these findings suggest that such an affective reaction may not be very common. In fact, the majority of participants reported only small shifts in their stress levels from pretravel to arrival, in both directions (i.e., mild stress and minor relief), which suggests that for most sojourners, relocating to a new culture may not be quite the “shock” or “euphoria” that some would expect.

In the control group sample we found little interindividual variation in stress trajectories over time. Indeed approximately two thirds of the control sample reported no shift from baseline levels of stress over time, while the remaining third was split between those showing an increase in stress over time relative to baseline and those showing a decrease in stress relative to baseline. Given that we have no additional information about the control group in terms of the life experiences they went through over the course of the study we cannot speculate as to the explanation for the deviations in stress in the third of the sample that did shift from baseline.

## Antecedents of Stress

Naturally, some of the variation in stress can be explained by individual difference variables. In this section we examine whether variations in personality and interpersonal reactivity (prior to the sojourn) can account for changes in sojourners’ stress. In addition, the role of cultural adaptation and coping strategies (during the sojourn) is also examined.

With regards to personality, previous research has found relations between adjustment and neuroticism, extraversion, conscientiousness, and agreeableness dimensions of personality (Swagler & Jome, 2005; Ward et al., 2004). Research in nonsojourning contexts has also demonstrated the buffering effects of extraversion, conscientiousness, agreeableness, and openness to experience for stress and the risk effect of neuroticism (e.g., Bowling & Eschleman, 2010; Cheung Chung et al., 2014; Matthews et al., 2006; Williams, Rau, Cribbet, & Gunn, 2009). We anticipate that the relationship between personality and stress in a sojourning context will be consistent with previous findings. Specifically, we expect that participants with higher levels of extraversion, conscientiousness, agreeableness, and openness will report lower levels of stress abroad than those with lower levels on these personality dimensions. Conversely, we anticipate that participants reporting higher levels of emotionality pretravel will report higher levels of stress abroad. With regards to honesty-humility, we expect that participants with lower levels on this factor will report higher levels of stress, due the known association between low levels of honesty and dysfunctional interpersonal traits (Holden et al., 2014; Lee & Ashton, 2005).

Research on the role of empathy in cultural adjustment has not previously separated perspective taking and empathetic concern components. However, based on more general research findings we anticipate that greater perspective taking will be associated with participants experiencing less or decreasing stress over time abroad (Galinsky et al., 2005). For empathetic concern and its association with stress we do not have any clear predictions, as previous research has found this factor to be associated with both desirable and undesirable interpersonal styles (Davis, 1983).

Cultural adaptation and its association with stress has been well documented. In this study however, we implement novel measures of sociocultural and psychological adaptation, the latter of which deviates from the typical operationalization by specifying psychological reactions specifically in relation to being in the host culture as opposed to context general psychological reactions (Demes & Geeraert, 2014). We anticipate that participants’ adaptation will be strongly associated with change in stress over time, with higher levels of adaptation relating to lower levels of stress and expect that participants in each of the different stress trajectories will be clearly distinguished by their levels of adaptation on arrival to the host country.

With regards to coping, approach strategies have been consistently associated with positive adjustment outcomes and avoidance strategies with negative ones (Herman & Tetrick, 2009; Ward & Kennedy, 2001). We expect therefore that a similar pattern of effects will emerge in our study such that approach and acceptance strategy use will be related to lower levels of stress, while avoidance, self-blame, and substance abuse will be related to higher levels of stress. Evidence in the field for the role of social support strategies in moderating stress has been less consistent than research on other coping strategies. Importantly however, such studies have failed to distinguish between support received from people locally (in the host country) from support received from people back home. This distinction is particularly relevant today, with the ease of communication across large geographical distances. In this study we do distinguish between close support (from people in the host country) and distant support (from people in the home country) and expect the former to be associated with lower stress and the latter with higher levels of stress. This would align with findings showing the detrimental effect of contact with home nationals while abroad (Geeraert et al., 2014).

We first use multilevel modeling to assess in the sojourner sample as a whole, the relationship between these explanatory variables and stress. Second, we employ multinomial logistic regression to examine differences in the explanatory variables across the five groups of participants experiencing different stress trajectories, as established in the earlier LCGA analysis. We expect the results of both of these analyses to be largely consistent with one another. Whereas the former analysis has the advantage of greater statistical and explanatory power, the latter allows for summarizing and describing the data in the form of profiles for distinct groups.

### Results

#### Explaining sojourners’ stress

The role of individual difference variables in moderating sojourners’ stress over time was examined through a series of longitudinal multilevel models. Independent sets of models were constructed for demographic variables, personality factors, interpersonal reactivity, cultural adaptation, and coping variables.^6^ Each model was built as a two-level model with time (t3 to t6) as the primary unit of analysis (Level 1) nested within individuals (Level 2).^7^ For each analysis, a first model was computed including time (t3 to t6) as a linear variable (coded 0 to 3), controlling for lagged stress.^8^ Grand-mean centered explanatory variables were added in the second model and the interaction of these variables with time added in the third model. As a comparison, and where appropriate, equivalent analyses were also carried out for the control group. All analyses were conducted following the procedures suggested by Hox (2010) and using MLwiN 2.30 (Rasbash, Browne, Healy, Cameron, & Charlton, 2014).

##### Demographics

The association between stress (t3 to t6) and a number of demographic variables was analyzed first. Eleven demographics were examined, including participant’s sex, nationality relative to their home country and parents, status of residence, number of languages spoken, and previous exposure to traveling, living abroad, and hosting international students. The explanatory ability of each demographic variable was examined separately in a two-level multilevel model. First, a basic model was constructed which included time and lagged stress (Model 1).^9^ The explanatory variable was then entered by itself (Model 2), followed by its cross-level interaction with time (Model 3). The results of the 11 multilevel models are summarized in Table 4. An effect of participant’s sex was found, such that male participants reported lower levels of stress than females while controlling for lagged stress and time. The number of languages spoken by participants was also related to stress, but only when controlling for the cross-level interaction with time. Speaking more languages was related to less stress but only at the start of the sojourn. Lastly, having previous travel experience was marginally but negatively associated with stress.[Table-anchor tbl4]

##### Personality

The role of pretravel personality (t1) on stress was modeled with time at the lowest level (*N*_*L1*_ = 6,648) nested within individuals (*N*_*L2*_ = 1,937). First, a basic model was constructed including a linear effect of time and the lagged stress variable (Model 1, *deviance* = 17,324.11, *df* = 5). The personality variables, as measured by the HEXACO, were then added to the model (Model 2, see Table 5) significantly improving the fit, χ^2^(6) = 299.42, *p* < .001. All six personality factors independently explained a significant amount of variance in sojourners’ stress. Specifically, sojourners reporting higher levels of honesty-humility, extraversion, agreeableness, and conscientiousness reported lower levels of stress while abroad. Higher levels of emotionality and openness to experience however were related to higher levels of stress abroad. Adding the cross-level interactions of time with each of the personality variables did not statistically improve the model (Model 3), χ^2^(6) = 4.36, *ns.*, nor did any of the individual interactions reach significance independently. As a set, these models suggest that the relationship between personality and stress was relatively stable over the course of the sojourn. The equivalent analysis was conducted for control participants, and revealed that stress was positively associated with emotionality and negatively associated with extraversion, agreeableness, and conscientiousness.^10^

##### Interpersonal reactivity

The relationship between the interpersonal reactivity variables and stress were also examined in a two-level model (*N*_*L1*_ = 6,648, *N*_*L2*_ = 1,937). As before, the basic model included a linear effect of time and the lagged stress variable (Model 1, *deviance* = 17,324.11, *df* = 5). The addition of empathetic concern and perspective taking (Model 2, see Table 6) improved the model fit, χ^2^(2) = 28.38, *p* < .001. Only perspective taking emerged as an independently significant explanatory variable however, indicating that sojourners who reported higher levels of perspective taking experienced less stress. The cross-level interactions of each variable with time did not significantly improve the model (Model 3, χ^2^ < 1). Thus, the relationship between stress and perspective taking did not change over time. In the equivalent analysis for control participants there was no effect of the interpersonal reactivity variables.^11^

##### Cultural adaptation

The association between both sociocultural and psychological adaptation with stress was examined next. Adaptation was operationalized as a time varying variable (situated at Level 1) in a two-level model (*N*_*L1*_ = 6,671, *N*_*L2*_ = 1,933). First, a basic model was constructed including the linear effect of time and the lagged stress variable (Model 1, *deviance* = 17,384.27, *df* = 5). Both psychological and sociocultural adaptation were added next (Model 2, see Table 7), improving the overall model fit, χ^2^(2) = 930.79, *p* < .001. Both types of adaptation were negatively related to stress. That is, higher levels of psychological and sociocultural adaptation were significantly associated with lower levels of stress overall. The interactions of adaptation with time (Model 3) further improved the model, χ^2^(2) = 178.41, *p* < .001, and the interaction between psychological adaptation and time emerged alongside the main effects of both types of adaptation. This interaction indicates that the relationship between psychological adaptation and stress is particularly pronounced at the earlier timewaves, but weakens somewhat over time.[Table-anchor tbl7]

##### Coping strategies

Sojourners’ coping strategies were measured at a single time point (at t4); thus, this variable was set as a time-invariant variable (at Level 2) in a two-level model looking at stress from t4 to t6 (*N*_*L1*_ = 4,774, *N*_*L2*_ = 1,788). First, a basic model was constructed including time and lagged stress (Model 1, *deviance* = 12,350.09, *df* = 5). The addition of the different coping strategies (Model 2, see Table 8) significantly improved model fit, χ^2^(7) = 313.39, *p* < .001. All coping strategies were independently significant explanatory variables in the model, with the exception of substance abuse. More specifically, whereas the approach, acceptance, and close support strategies were all associated with lower levels of stress; the avoidance, self-blame, and distant support strategies were associated with higher levels of stress. Interestingly, and as predicted, whereas support in the host country (close support) was associated with lower levels of stress, support from the home country (distant support) was associated with higher levels of stress.[Table-anchor tbl8]

Adding the cross-level interactions between coping strategies and time significantly improved the model (Model 3), χ^2^(7) = 96.38, *p* < .001. Controlling for these interactions, the substance abuse strategy now emerged as being significantly associated with stress. Importantly, however, the associations between stress and the coping strategies were qualified by time. This was the case for all strategies, except approach coping. The relationship between stress and these coping strategies was generally stronger at the start of the sojourn than at the later waves. The equivalent analyses for control participants revealed that stress was positively associated with avoidance, substance abuse, and self-blame coping, and negatively associated with approach and acceptance coping.^12^

##### Composite model

Having examined the explanatory ability of demographics, personality, interpersonal reactivity, cultural adaptation, and coping strategies on stress in separate model sets, we decided to bring all of these together into a single composite model. Specifically, variation in stress was examined in a two level model looking at stress from t4 to t6 (*N*_*L1*_ = 4,398, *N*_*L2*_ = 1,607). A basic model was constructed including time and lagged stress (*deviance* = 11,386.55, *df* = 5). In the composite model, personality, interpersonal reactivity, cultural adaptation, coping, and demographics (sex, previous travel experience, and number of spoken languages) were added, as well as the previously significant cross-level interactions for cultural adaptation, coping, and number of languages spoken, together improving model fit, χ^2^(30) = 713.16, *p* < .001.

The composite model makes it possible to examine the explanatory ability of individual variables while controlling for all other variables. Consistent with the earlier demographics analysis, there was a significant effect of sex, prior travel experience, and the interaction between time and number of languages spoken. For personality, a negative association with stress remained for extraversion, and a positive association for emotionality and openness to experience. There were no significant effects for the interpersonal reactivity variables. For cultural adaptation, the only effect to remain was the negative relationship between psychological adaptation and stress. In contrast to the earlier analysis, time no longer qualified this effect of adaptation. In terms of coping, the relationship with stress was positive (associated with high stress) for avoidance, self-blame, substance abuse, and distant support, and negative (associated with low stress) for acceptance, and close support. As before all of these effects were qualified by time, with the exception of approach coping, such that the association between coping strategy use and stress was strongest at the earlier than later timewaves.

#### Explaining sojourners stress trajectories

Above we established the explanatory role of a number of key individual difference variables in accounting for variations in stress over the course of the sojourn across the sojourner sample as a whole (variable-centered approach). In this section we examine these associations in a different way, by exploring individual differences between participants belonging to the five distinct stress trajectory groups. Specifically, we examined whether membership to these trajectory groups could be accounted for by individual differences in variables measured both before traveling and after arrival to the host country. Using multinomial logistic regression, with class membership as a categorical dependent variable, we examined whether individual differences in personality, interpersonal reactivity, cultural adaptation, and coping strategies could account for the likelihood of experiencing certain stress and adjustment trajectories, controlling for baseline stress. Where a particular explanatory variable is found to significantly improve model fit, we inspected class comparisons to determine how the likelihood of experiencing different stress trajectories differs as a function of the variable. Separate sets of analyses were conducted for personality, interpersonal reactivity, cultural adaptation, and coping variables (see Table 9).[Table-anchor tbl9]

##### Personality

In the personality model, emotionality, χ^2^ = 14.80, *p* = .005, and extraversion, χ^2^ = 41.09, *p* < .001, were found to be significant explanatory variables for stress trajectory membership while controlling for all other personality factors and baseline stress. An examination of differences across classes indicates that with higher levels of emotionality at baseline, participants were more likely to experience a reverse J-curve pattern of stress while abroad. With higher levels of reported extraversion however, participants were more likely to experience a resilience pattern of stress over all other patterns. A minor relief pattern of stress was also more likely than a mild stress, reverse J-curve or inverse U-curve pattern for participants with higher levels of extraversion.

##### Interpersonal reactivity

In the interpersonal reactivity model, empathetic concern, χ^2^ = 17.17, *p* < .005, but not perspective taking, χ^2^ = 8.42, *p* = .08, emerged as an independently significant explanatory variable for stress trajectory membership over and above perspective taking and baseline stress. Comparisons of stress trajectory classes revealed that with higher reported empathetic concern pretravel, the more likely participants were to experience a resilience pattern of stress over a minor relief, mild stress, or inverse U-curve pattern of stress. Mild stress and reverse J-curve patterns were also more likely to be experienced than inverse U-curve patterns in participants with higher levels of empathetic concern pretravel.

##### Cultural adaptation

In the cultural adaptation model both sociocultural, χ^2^ = 29.39, *p* < .001, and psychological adaptation, χ^2^ = 251.00, *p* < .001, were independently significant explanatory variables for stress trajectory membership when entered with baseline stress. Participants were found to be more likely to experience a reverse J-curve pattern of stress than any other pattern the lower their psychological adaptation was on entry to the host country. Participants with low sociocultural adaptation were also more likely to experience a reverse J-curve pattern of stress than a mild stress, minor relief, or resilience pattern. With higher levels of psychological adaptation, a resilience pattern was significantly more likely than any other stress trajectory. With higher levels of sociocultural adaptation a resilience pattern was more likely than all others but the minor relief pattern.

##### Coping strategies

In the coping model, all but two of the coping strategies (approach and substance abuse) made an independently significantly contribution to the model, controlling for baseline stress, that is, avoidance, χ^2^ = 74.54, *p* < .001, acceptance, χ^2^ = 39.76, *p* < .001, close support, χ^2^ = 26.18, *p* < .001, distant support, χ^2^ = 50.30, *p* < .001, and self-blame χ^2^ = 86.11, *p* < .001, although the approach strategy was marginally significant according to our criteria, χ^2^ = 13.52, *p* = .009. Notably, reverse J-curve and inverse U-curve patterns were found to be more likely than other patterns in participants reporting greater use of avoidance strategies, self-blame, and support from people back home. In contrast, participants were found to be more likely to experience a resilience trajectory than any other the more they employed acceptance coping and close support. In the case of close support, however, a minor relief pattern was just as likely as a resilience pattern.

##### Composite model

Finally, a person-centered composite model was conducted and was found to largely replicate the variable-centered findings. Specifically, controlling for all other variables, psychological adaptation (χ^2^ = 130.09, *p* < .001) and the three coping strategies, avoidance (χ^2^ = 40.22, *p* < .001), acceptance (χ^2^ = 20.18, *p* < .001), and self-blame (χ^2^ = 49.29, *p* < .001) made independently significant contributions to the model. However, none of the personality variables remained independently significant in the person-centered composite model. That none of the personality variables are independently significant in this model in contrast to the variable-centered analysis may be due to the relatively weaker statistical power of the person-centered analysis.

### Discussion

In this section we examined associations between sojourners stress over time and personality, interpersonal reactivity, cultural adaptation, and coping strategies as potential moderators of stress. These associations were examined through both a variable-centered (longitudinal multilevel modeling) and a person-centered approach (multinomial logistic regression). Although each type of analysis address the same research question, they also provide a different perspective on the data, the former a more statistically powerful and explanatory approach and the second a more descriptive “profiling” approach. Despite the differences between these two methods, the conclusions we can draw from their results are largely convergent.

With regards to personality, our results were consistent with predictions and previous research (Cheung Chung et al., 2014; Swagler & Jome, 2005; Ward et al., 2004). Emotionality was found to be associated with overall increased levels of stress and stress trajectories characterized by peaks in stress. Extraversion had a buffering effect, such that participants with higher levels on this variable tended to report lower levels of stress in general and stress trajectories characterized by drops in stress relative to pretravel. In addition, honesty-humility, agreeableness, and conscientiousness were also found to be associated with lower overall levels of stress, although this only materialized in the variable-centered analyses—probably due to the increased statistical power of analyzing the sample as a whole, rather than as split into groups. Although low levels of honesty have been linked with dysfunctional interpersonal traits (Holden et al., 2014; Lee & Ashton, 2005), the negative relationship between honesty-humility and stress in a cross-cultural transition has not been demonstrated before. Finally, and contrary to predictions, openness to experience was found to be associated with increased levels of stress. One speculative explanation may be that sojourners with greater openness to experience are more likely to put themselves in novel situations that they may find challenging or stressful. Further, openness to experience is believed to produce the most inconsistent results of all the main personality dimensions. For instance, there is a well-established link between personality and subjective well-being for all factors of the Big Five, with the exception of openness (Steel, Schmidt, & Shultz, 2008). Similarly, openness is the only factor of the Big Five that does not link well to mental well-being (Malouff, Thorsteinsson, & Schutte, 2005). Thus, it is difficult to interpret our findings with the openness factor in this case.

With regards to the interpersonal reactivity variables, in the variable-centered analysis perspective taking was found to be significantly associated with lower stress, while empathetic concern was not. The reverse was found for the person centered analysis in that empathetic concern was significantly associated with trajectories with drops in stress but perspective taking was not. This might imply that these associations may not be as stable or robust. Indeed, both effects disappeared when controlling for other factors such as emotionality or extraversion.

In both types of analysis sociocultural and psychological adaptation were found to be associated with stress. Psychological adaptation was revealed to have a particularly powerful association with stress even when controlling for all other explanatory variables, suggesting that feeling psychologically adapted to the culture is strongly tied to sojourners perception of stress while abroad. Interestingly, the association between psychological adaptation and stress was more pronounced at the earlier waves compared with the later waves. This is understandable as sojourners experience a number of significant life changes in the first weeks of their sojourn. Later on, after the sojourner has become more accustomed to the host culture, those factors influencing stress may be less concerned with cultural adjustment.

The coping strategies that participants employed while abroad was found to be highly relevant to stress across all types of analyses. In line with predictions and previous research (Herman & Tetrick, 2009; Wang et al., 2012; Ward & Kennedy, 2001), participants were found to report lower levels of stress overall or to experience stress trajectories characterized by decreases in stress when relying on approach, acceptance, and close support strategies. Conversely, higher levels of stress were related to greater use of avoidance, self-blame, and distant support strategies. A marginal effect of substance abuse was also found, suggesting that greater use of substances as a means to cope is maladaptive and related to higher levels of stress abroad.

It is worth noting the findings reported in this section could only be revealed through the longitudinal design of this study. Specifically, the association between personality variables recorded pretravel remained stable over time, that is, the relationship was the same at both the later and earlier timewaves. This was not the case for psychological adaptation and coping strategies, whose associations with stress were most marked at the earlier waves and then diminished over time.

## Consequences of Stress

The final series of analyses involves examining the relationship between stress levels and stress trajectory group membership and two key behavioral indicators of (mal)adjustment: number of family changes and early return. In many cases, moving host family is a sign of failure to adjust to the new family environment and while most participants stay with one family for the duration of their exchange, some do move families multiple times. Similarly, most participants stay for the full term of their intercultural exchange but some sojourners, experiencing difficulties in adjustment, return home early. It is anticipated that incidence of family change and early return will vary as a function of stress during the exchange. Specifically, participants reporting higher levels of stress relative to baseline or those belonging to groups characterized by increases in stress on entry to the host country are expected to be more likely to change host families and to return home early than those characterized by decreases in stress or stable stress.

### Results

#### Family change

The variable family change provides a count of the number of families a sojourner has stayed with. A variable resulting from such a *counting process* has a Poisson distribution,^13^ and thus the data was analyzed by means of a Poisson regression. Average stress over the duration of the sojourn (t3 to t6) was computed for each sojourner and then entered in to the regression model with the family change variable as the dependent variable. Results of the Poisson regression indicated that sojourners’ average stress level was indeed positively associated with family change (*B* = .21, Wald’s χ^2^ = 27.63, *p* < .001), such that sojourners with higher stress levels were more likely to change family. Importantly, the effect of average stress also remained (*B* = .30, Wald’s χ^2^ = 39.70, *p* < .001) when controlling for sojourners’ baseline stress (at t1).

We next examined the role of coping strategies in the occurrence of family change by means of a Poisson regression. Specifically, family change was regressed on coping strategies and the regression model was significant, χ^2^(7) = 57.46, *p* < .001. There was a negative relationship between the number of family changes and acceptance (*B* = −.12, Wald’s χ^2^ = 7.28, *p* < .01) and close support coping (*B* = −.07, Wald’s χ^2^ = 5.18, *p* = .023). Interestingly, a positive relationship emerged between family change and approach (*B* = .24, Wald’s χ^2^ = 21.11, *p* < .001) and distance support coping (*B* = .12, Wald’s χ^2^ = 22.77, *p* < .001). Apart from close support coping, these relationships remained when controlling for average levels of stress.

Finally, we examined the relationship between stress trajectories and family change, by means of a Poisson regression. The stress trajectories were regressed on family change and the overall model was significant, χ^2^(4) = 38.14, *p* < .001. Pairwise comparisons revealed that individuals in the mild stress (*M* = 1.41, *SD* = .74) and minor relief (*M* = 1.36, *SD* = .71) groups lived with significantly fewer families on average than those in the reverse J-curve (*M* = 1.61, *SD* = .96) and inverse U-curve (*M* = 1.61, *SD* = .96) groups (all *p’s* < .05). The resilience group was found to have changed host families the least often of all groups, that is, they lived with significantly fewer families overall (*M* = 1.17, *SD* = .46, all *p’s* < .001). Finally, the reverse J-curve and inverse U-curve groups did not differ from one another, nor did the mild stress and minor relief groups from one another.

#### Early return

The event of early sojourn termination was analyzed by means of a binary logistic regression. In the entire sample, we had an early return rate of 4%.^14^ Early return was regressed on average stress level for the duration of the sojourn (t3 to t6). This model was significant, Wald χ^2^(1) = 26.27, *p* < .001. An odds ratio of 1.91 indicated that for each one unit step along the stress scale, the odds of early return almost double. For instance, participants with an average stress score at the 10th percentile have a 1.7% risk of early return, compared with a 6.6% risk for participants at the 90th percentile for stress. Crucially, this pattern of results was identical when controlling for baseline stress.

The relationship between the number of family changes and early return was examined next by means of a logistic regression. As expected, the analysis showed that family change was positively associated with early return, Wald χ^2^(1) = 25.79, *p* < .001. An odds ratio of 1.85 indicated that for each additional family change the odds of early return nearly double. For instance, sojourners who remained with a single host family (*N* = 1,448, 71.9%), had a 2.8% risk of early return. However, sojourners who changed family once (*N* = 395, 19.6%) had a 5.1% risk, and this increased to 9.0% for two changes (*N* = 134, 6.7%) and 15.5% for three changes (*N* = 27, 1.3%). These results were also replicated when controlling for both average and baseline stress.

Finally, we examined the incidence of early return across the stress trajectories using binary logistic regression. The overall contribution of stress trajectories on the prediction of early return was significant, χ^2^(4) = 37.33, *p* < .001. Comparisons of the groups revealed that participants who experienced a reverse J-curve or inverse U-curve pattern of stress and adjustment were significantly more likely to return home early than participants who experienced a mild stress, minor relief, or resilience pattern. Individuals reporting a resilience pattern had significantly lower early return rates than individuals in all of the other groups. Specifically, 17.2% of the reverse J-curve participants and 13.3% of the inverse U-curve participants returned home early, while only 3.7% of the mild stress group, 2.7% of the minor relief group, and 1.2% of the resilience group did so. All other comparisons were not significant, that is early return rates did not differ significantly between the reverse J-curve and inverse U-curve groups, nor between the mild stress and minor relief groups.

### Discussion

Overall, the results of the early return and family change analyses demonstrate that average levels of stress while abroad are significantly associated with family changes and early return rate. In addition, the types of coping strategies that participants employed were also related to family changes, such that greater use of acceptance coping and seeking support from people in the host country was related to fewer family changes and greater use of approach coping and seeking support from people in the home country was related to more family changes. While it would be interesting to look at the association between coping and early return, statistical limitations (due to the small proportion of students returning home early) means this was not possible.

With regards to the stress trajectories and their relationship with family change and early return, the pattern of findings seem to suggest that there are three distinguishable classes in relation to these variables. Overall, the resilient group appears to differ from all others, the J and U-curve classes differ from all others but not from each other and likewise the mild stress and minor relief groups differ from all others but not from each other.

## General Discussion

In this article we built upon a large body of theory and research regarding the stress and adjustment patterns of intercultural travelers, their antecedents, and consequences (Hechanova-Alampay et al., 2002; Herman & Tetrick, 2009; Swagler & Jome, 2005; Zhang & Goodson, 2011). We presented a series of analyses from a longitudinal study of approximately 2,500 intercultural exchange students who traveled from over 40 different countries and spent a year abroad in one of over 50 different host destinations. Taking advantage of the longitudinal and multinational nature of this study and the presence of a control group, we examined a number of central research questions to the acculturation field. The results of these examinations present a number of theoretical contributions which are discussed below.

### Stress Over Time

In this study we recorded stress on multiple occasions over time, in both a sojourning and a control group. This design enabled us to examine whether sojourners typically experience greater variation in stress over time than nonsojourning peers. Indeed we found that sojourners’ experienced significant variation in stress over time while a control group did not. This highlights the uniqueness of the sojourn in terms of its psychological impact and supports the examination of the nature of this impact as an important area of enquiry.

Researchers have long attempted to identify a “standard” temporal pattern to describe the cultural stress and adjustment of intercultural travelers (Geeraert & Demoulin, 2013; Oberg, 1960; Ward et al., 1998). In this article we demonstrated that a single overarching description of the course of adjustment over time may be inadequate and instead were able to identify five patterns which described the adjustment of different subsets of sojourners. We detected a number of patterns that have been observed previously, such as an inverse U-curve (Hechanova-Alampay et al., 2002), reverse J-curve (Ward et al., 1998), and resilience pattern (Geeraert & Demoulin, 2013), in addition to other patterns not previously observed in this context. This highlights that reactions to an intercultural relocation are not uniform and that earlier theoretical and methodological approaches have not captured this.

Further, theorists have proposed that relocating to a new culture will inevitably lead to stress and strain (Oberg, 1960) but our research shows that this is not always the case. Indeed, the large majority of our sample, those in the mild stress or minor relief groups appeared only to be marginally affected by the move, at least in terms of their stress levels. It is likely that the unique characteristics of this particular population are responsible for the more typical low stress reaction, as the whole experience is intended to be an opportunity for personal growth, learning, and to expand cultural awareness (Maddux & Galinsky, 2009; Montuori & Fahim, 2004). It would be interesting therefore to see what patterns emerge in different sojourning or immigrant samples, with the application of such person-centered analyses. We would expect similar trajectories as found in this study to emerge, but that the proportion of individuals experiencing the different patterns would vary. In an immigrant sample for example, a greater proportion of individuals may be expected to experience inverse U-curve or reverse J-curve patterns characterized by substantial peaks in stress, as the relocation experience of immigrants involves relatively more personal, emotional, and financial upheaval than that experienced by exchange students.

### Antecedents of Stress Trajectories

Following the identification of different stress reactions to the relocation we examined the role of personality, empathy, cultural adaptation, and coping strategies in moderating stress over time. Looking at personality alone, the results suggest a buffering effect on stress of honesty-humility, extraversion, and conscientiousness but a risk effect of emotionality. Similarly, for the interpersonal reactivity variables alone, perspective taking was shown to be adaptive in the variable-centered analysis and empathetic concern in the person-centered analysis. The fact that alternative variables were found to be significant across the two types of analysis may suggest that perspective taking is more powerful in explaining variation in stress levels in general over the sojourn but that empathetic concern is related more to the particular course of increases or decreases in stress over time, that is its contribution to stress may be less uniform over time. For the coping variables alone, their associations with stress were found to vary over time, such that the adaptive role of acceptance coping and seeking support from people in the host country, and the maladaptive role of avoidance, seeking support from people back home, and self-blame were particularly pronounced in the early waves, but less so as the sojourn progressed.

Controlling for all explanatory variables revealed robust and stable effects of emotionality, extraversion, psychological adaptation, avoidance coping, acceptance coping, seeking support from locals and self-blame. Robust time varying effects were found for both close and distant support and self-blame. Interestingly, the relationship between support and stress was different depending on the proximity of social support. Seeking support from people remotely back in the home country was related to having higher stress in general, while seeking support from people local in the host country was related to having lower stress in general. This finding is also in line with previous research showing that too much contact with home nationals can hinder cultural adjustment (Geeraert et al., 2014).

Overall, these findings are consistent with theory and previous research (Carver et al., 1989; Davis, 1983; Galinsky et al., 2005; Herman & Tetrick, 2009; Lazarus & Folkman, 1984; Skinner et al., 2003; van Oudenhoven & van der Zee, 2002; Ward & Kennedy, 1999; Ward & Kennedy, 2001). However, this research provides a valuable contribution to the literature by demonstrating these effects across a multinational sojourner sample (from both a sending and hosting perspective) and using longitudinal data which has enabled us to demonstrate the robustness and stability of these associations over time.

### Consequences of Stress Trajectories

Lastly, we found that different patterns of stress over the exchange were related to key behavioral indicators of adjustment, with higher levels of stress relating to increased likelihood of changing host families and returning home early. The types of coping strategies that sojourners used were also found to be associated with family changes. Interestingly, the approach strategy was related to more family changes, which may be because participants using the approach strategy took action when experiencing difficulty adjusting to one family and thus moved to another. Altogether these results are particularly meaningful as we were able to record participants’ actual behavior; whether they did indeed return home early and how many times they did change host family. Having behavioral measures make these findings particularly strong and novel. Further, while research on international assignees has previously examined precursors to early return, these studies have typically only utilized measures of desire or intention to leave rather than actual behavior (Black & Gregerson, 1990; Caligiuri, 2000).

### Strengths and Limitations

This study has a number of important strengths that make this study quite unique in terms of its design and research findings. First of all, the fact that this is a longitudinal study is crucial, as this allowed us to monitor and examine the stress and adjustment patterns of sojourners over the entire course of their 8–10 month intercultural exchange while also controlling for baseline, pretravel levels of stress. We were also able to examine the antecedents and consequences of stress and make claims regarding their temporal variability or stability.

Certainly this is not the first study to examine sojourner adjustment over time; but it is the first of its kind to benefit from such a culturally diverse sample of participants. Specifically, this sample included participants traveling from over 40 different countries and traveling to one of 51 different destinations. Naturally, this cultural diversity summons the question of cultural distance, and its role in stress (Babiker, Cox, & Miller, 1980). Cultural distance is a multifaceted construct, which encompasses economic factors (i.e., GDP), geographical features (i.e., climate), sociocultural aspects (i.e., ethnicity, language), differences in psychological values and dimensions (i.e., individualism-collectivism, universalism, tightness-looseness, etc.), as well as subjective perceptions of cultural distance (Fearon, 2003; Gelfand et al., 2011; Hofstede, 2001; Kogut & Singh, 1988; Schwartz et al., 2012; Searle & Ward, 1990; Van de Vliert, 2009). Indeed, this dataset is ideally suited to address the cultural distance puzzle, and we have explored this extensively elsewhere. Our analyses show that both subjective and more objective measures of cultural distance do impact on measures of acculturative stress (Geeraert & Demes, in preparation). Still, the diversity of this sample does mean that we can generalize these findings across cultures more confidently than has been possible in previous studies that examined acculturation from a single sending or single hosting perspective (Hechanova-Alampay et al., 2002; Ward et al., 1998; Wang et al., 2012).

What is less clear is how well the findings reported in this article would generalize to other acculturating groups such as expatriates, immigrants, or refugees. These groups differ along a number of important dimensions such as whether their relocation is voluntary or not and whether it is permanent or temporary (Berry, 1990). Also, our exchange students, placed with local host families and attending local schools may have more day to day contact with host nationals than individuals in other acculturating groups. Research has shown that contact with host nationals is important to cultural adjustment (Geeraert et al., 2014; Zhang & Goodson, 2011) and this may explain at least in part, why we found a number of positive adjustment reactions to the exchange (e.g., minor relief and resilience). In other groups who have less frequent contact with host nationals, patterns of stress and adjustment may differ. It would be interesting therefore, to explicitly test this by replicating the current study in these different samples.

In terms of the statistical approach to the analyses reported here, in utilizing both variable and person-centered techniques we were able to provide different perspectives on the course of stress and adjustment over time. Specifically we were able to offer both explanatory and descriptive findings from the data. The descriptive approach provided a novel perspective on sojourner adjustment over time relative to previous approaches (Hechanova-Alampay et al., 2002; Ward et al., 1998). We argued that identifying multiple patterns of stress over time recognizes and demonstrates that not all individuals react in the same way to an intercultural relocation. The unique features of sojourners reacting in different ways can therefore be examined and provide a more tailored view of the acculturation process and the factors that may facilitate or hinder it (see also Wang et al., 2012).

With regards to the findings on coping strategy use, these have a high level of practical relevance as they can be used to inform interventions and training programs for relocating individuals. While some level of stress may be inevitable for sojourning groups, this research suggests that employing certain coping strategies over others may ameliorate the degree and or duration of this stress.

## Conclusion

The body of research on acculturation is growing rapidly as intercultural contact becomes an increasingly common experience for individuals around the world. This phenomenon is closely related to advances in technology which make cross-cultural travel and communication so easy now. It is important for research to keep up with this change by exploring and understanding how different groups of individuals experience and react to this. This study has been the first of its kind to examine acculturation in such a culturally diverse sample of sojourners in conjunction with a longitudinal design and control group. More research of this scope and nature is needed in order to better understand the acculturation experience and how key variables interact to influence this experience over time.

## Figures and Tables

**Table 1 tbl1:** Sample Size (N) per Country Travelled From (Sending) and Country Travelled to (Hosting), Ordered Alphabetically

Country travelled	From	To
Argentina	16	101
Australia	9	31
Austria	66	22
Belgium	64	77
Bolivia	5	14
Brazil	114	75
Canada	14	60
Chile	69	37
China	68	63
Columbia	23	6
Costa Rica	30	54
Czech Republic	11	17
Denmark	31	62
Dominican Republic	11	36
Ecuador	13	21
Egypt	0	5
Finland	61	41
France	76	59
Germany	279	255
Ghana	12	3
Honduras	6	19
Hong Kong	37	6
Hungary	17	25
Iceland	3	14
India	20	12
Indonesia	43	3
Italy	403	118
Japan	94	91
Kenya	0	1
Latvia	0	5
Malaysia	10	16
Mexico	29	23
Netherlands	1	24
New Zealand	66	61
Norway	112	52
Panama	6	31
Paraguay	11	15
Peru	2	22
Philippines	3	6
Portugal	4	23
Russia	8	31
South Africa	0	17
Spain	14	15
Sweden	14	24
Switzerland	68	70
Thailand	333	28
Tunisia	0	3
Turkey	53	14
USA	137	655
Venezuela	14	17

**Table 2 tbl2:** Fit Indices for Latent Class Growth Analysis on Sojourners’ Perceived Stress

	Two-class model		Three-class model		Four-class model		Five-class model		Six-class model	
	linear	quadratic	linear	quadratic	linear	quadratic	linear	quadratic	linear	quadratic
Free parameters	9	11	12	15	15	19	18	23	21	27
Log likelihood	−9,186.00	−9,166.37	−8,998.92	−8,970.25	−8,937.15	−8,906.17	−8,907.75	−8,867.46	−8,883.53	−8,844.40
BIC	18,440.47	18,416.43	18,089.14	18,054.63	17,988.42	17,956.90	17,952.44	17,903.90	17,926.84	17,894.23
aBIC	18,411.87	18,381.48	18,051.01	18,006.97	17,940.76	17,896.53	17,895.25	17,830.83	17,860.12	17,808.45
Entropy	.56	.56	.71	.71	.68	.68	.64	.70	.63	.71
LMR-LRT *p*	<.001	<.001	<.001	<.001	.008	.03	.21	.16	.07	.12
BLRT *p*	<.001	<.001	<.001	<.001	<.001	<.001	<.001	<.001	<.001	<.001
Class counts (fit probabilities)										
c#1	909 (.86)	911 (.86)	489 (.84)	468 (.77)	110 (.84)	100 (.85)	128 (.81)	819 (.76)	59 (.84)	86 (.87)
c#2	1,106 (.88)	1,104 (.87)	137 (.83)	151 (.70)	1016 (.83)	113 (.68)	78 (.86)	83 (.87)	193 (.71)	45 (.73)
c#3			1,389 (.88)	1,396 (.93)	807 (.78)	816 (.79)	866 (.78)	951 (.82)	327 (.74)	75 (.79)
c#4					82 (.87)	86 (.73)	850 (.71)	64 (.75)	239 (.69)	765 (.75)
c#5							93 (.72)	98 (.81)	1147 (.72)	979 (.82)
c#6									50 (.77)	65 (.69)
*Note*. BIC = Bayesian Information Criteria; aBIC = sample size adjusted BIC; LMR-LRT = Lo-Mendell-Rubin Likelihood Ratio Test; BLRT = Bootstrap Likelihood Ratio Test; c# = class number.										

**Table 3 tbl3:** Fit Indices for Latent Class Growth Analysis on Controls’ Perceived Stress

	Two-class model		Three-class model		Four-class model		Five-class model	
	linear	quadratic	linear	quadratic	linear	quadratic	linear	quadratic
Free parameters	9	11	12	15	15	19	18	23
Log likelihood	−1,736.45	−1,733.90	−1,697.15	−1,690.57	−1,686.35	−1,677.97	−1,682.21	−1,670.57
BIC	3,527.81	3,534.93	3,467.52	3,472.67	3,464.24	3,471.89	3,474.26	3,481.51
aBIC	3,499.25	3,500.02	3,429.44	3,425.07	3,416.63	3,411.59	3,417.14	3,408.52
Entropy	.52	.52	.69	.70	.71	.68	.70	.70
LMR-LRT *p*	.005	.02	<.005	.008	.03	.03	.23	.61
BLRT *p*	<.001	<.001	<.001	<.001	<.001	<.001	.05	.01
Class counts (fit probabilities)								
c#1	213 (.84)	195 (.83)	47 (.84)	85 (.85)	7 (.89)	108 (.82)	6 (.86)	32 (.88)
c#2	234 (.85)	252 (.86)	88 (.84)	45 (.86)	287 (.84)	39 (.87)	289 (.81)	245 (.79)
c#3			312 (.88)	317 (.87)	108 (.82)	293 (.91)	49 (.78)	9 (.88)
c#4					45 (.83)	7 (.82)	7 (.90)	12 (.85)
c#5							96 (.81)	149 (.77)
*Note*. BIC = Bayesian Information Criteria; aBIC = sample size adjusted BIC; LMR-LRT = Lo-Mendell-Rubin Likelihood Ratio Test; BLRT = Bootstrap Likelihood Ratio Test; c# = class number.								

**Table 4 tbl4:** Multilevel Analysis of Demographic Predictors on Stress

	Effect of IV			Effect of IV × Time			Model statistics		
IV	*B*	*SE*	*p*	*B*	*SE*	*p*	Deviance	χ^2^(1)	*p*
Male^a^									
Model 2	−.081	.024	.001	—	—	—	17,641.48^b^	10.82	.001
Model 3 (+ time)	−.084	.039	.033	.003	.022	.905	17,641.47	.01	.920
Nationality of participant different from the home country^a^									
Model 2	.043	.046	.353	—	—	—	17,651.43^b^	.87	.351
Model 3 (+ time)	.110	.076	.148	−.046	.042	.268	17,650.21	1.22	.269
Nationality of participant different from nationality of either parent^a^									
Model 2	.034	.030	.262	—	—	—	17,651.04^b^	1.26	.262
Model 3 (+ time)	.037	.049	.450	−.002	.027	.942	17,651.04	.00	1.000
Nationality of one parent different from nationality of other parent^a^									
Model 2	.022	.042	.606	—	—	—	17,652.03^b^	.27	.603
Model 3 (+ time)	.113	.067	.093	−.065	.038	.083	17,649.02	3.01	.083
Has participant lived in home country since birth^a^									
Model 2	.031	.048	.521	—	—	—	16,438.88^c^	.42	.517
Model 3 (+ time)	−.008	.076	.915	.028	.042	.513	16,438.46	.42	.517
Number of languages spoken by participant									
Model 2	−.011	.011	.310	—	—	—	16,438.26^c^	1.04	.308
Model 3 (+ time)	−.064	.018	.000	.037	.010	.000	16,424.75	13.51	.000
Has participant previously travelled abroad^a^									
Model 2	−.077	.031	.013	—	—	—	16,433.11^c^	6.19	.013
Model 3 (+ time)	−.064	.049	.193	−.009	.028	.742	16,433.00	.11	.740
Did participant previously sojourn^a^									
Model 2	.014	.035	.685	—	—	—	17,652.13^b^	.17	.680
Model 3 (+ time)	.007	.057	.902	.005	.032	.871	17,652.11	.02	.888
Number of months participant previously sojourned									
Model 2	−.002	.005	.778	—	—	—	17,652.22^b^	.08	.777
Model 3 (+ time)	−.005	.009	.525	.003	.005	.555	17,651.87	.35	.554
Has a member of family previously sojourned^a^									
Model 2	.000	.023	.994	—	—	—	16,439.30^c^	.00	1.000
Model 3 (+ time)	.008	.037	.836	−.005	.020	.797	16,439.23	.07	.791
Has the participants’ family previously hosted an exchange student^a^									
Model 2	−.025	.027	.352	—	—	—	16,438.43^c^	.87	.351
Model 3 (+ time)	.049	.044	.269	−.052	.024	.033	16,433.88	4.55	.033
^a^ Variable coded 1 (yes) or 0 (no). ^b^ Compared with Model 1, *N*_*L1*_ = 6,770, *N*_*L2*_ = 2,015, *deviance* = 17,652.30, *df* = 5. ^c^ Compared with Model 1, *N*_*L1*_ = 6,297, *N*_*L2*_ = 1,845, *deviance* = 16,439.30, *df* = 5.									

**Table 5:** Multilevel Analysis of Personality Factors on Stress

	Model 2			Model 3		
	(personality)			(personality + time)		
	*B*	*SE*	*p*	*B*	*SE*	*p*
Intercept	3.079	.017	.000	3.079	.017	.000
Time	−.108	.010	.000	−.108	.010	.000
Stress lag	.447	.012	.000	.447	.012	.000
Predictors						
Honesty-humility	−.043	.013	.001	−.039	.021	.066
Emotionality	.099	.013	.000	.110	.020	.000
Extraversion	−.186	.014	.000	−.177	.022	.000
Agreeableness	−.035	.014	.014	−.059	.022	.009
Conscientiousness	−.054	.013	.000	−.045	.020	.027
Openness	.030	.014	.027	.008	.022	.695
Interactions						
Time × Honesty-humility				−.003	.012	.822
Time × Emotionality				−.008	.011	.486
Time × Extraversion				−.007	.012	.578
Time × Agreeableness				.017	.012	.172
Time × Conscientiousness				−.006	.011	.570
Time × Openness				.015	.012	.195
Residual variance						
σ_*e*_^2^ (L1: time)	.746	.015	.000	.745	.015	.000
σ_*u*_^2^ (L2: individual)	.012	.008	.141	.012	.008	.134
Model statistics						
*Deviance (df)*	17,024.69 (11)			17,020.33 (17)		
χ^2^ *(df)*	299.42 (6)			4.36 (6)		
*p*	.000			.628		

**Table 6:** Multilevel Analysis of Interpersonal Reactivity on Stress

	Model 2			Model 3		
	(interpersonal)			(interpersonal + time)		
	*B*	*SE*	*p*	*B*	*SE*	*p*
Intercept	3.084	.018	.000	3.084	.018	.000
Time	−.112	.010	.000	−.112	.010	.000
Stress lag	.539	.011	.000	.539	.011	.000
Predictors						
Perspective taking	−.063	.014	.000	−.074	.023	.002
Empathic concern	−.020	.014	.163	−.026	.023	.268
Interactions						
Time × Perspective taking				.008	.013	.548
Time × Empathic concern				.004	.013	.747
Residual variance						
σ_*e*_^2^ (L1: time)	.790	.014	.000	.790	.014	.000
σ_*u*_^2^ (L2: individual)	.000	.000	—	.000	.000	—
Model statistics						
*Deviance (df)*	17,295.73 (7)			17,295.04 (9)		
χ^2^ *(df)*	28.38 (2)			.69 (2)		
*p*	.000			.709		

**Table 7 tbl7:** Multilevel Analysis of Adaptation Variables on Stress

	Model 2			Model 3		
	(adaptation)			(adaptation + time)		
	*B*	*SE*	*p*	*B*	*SE*	*p*
Intercept	3.054	.017	.000	3.051	.017	.000
Time	−.091	.009	.000	−.093	.009	.000
Stress lag	.269	.012	.000	.278	.012	.000
Predictors						
Psychological adaptation	−.331	.014	.000	−.490	.021	.000
Sociocultural adaptation	−.134	.014	.000	−.155	.021	.000
Interactions						
Time × Psychological adaptation				.114	.011	.000
Time × Sociocultural adaptation				.018	.011	.121
Residual variance						
σ_*e*_^2^ (L1: time)	.622	.013	.000	.596	.012	.000
σ_*u*_^2^ (L2: individual)	.078	.009	.000	.089	.009	.000
Model statistics						
*Deviance (df)*	16,453.48 (7)			16,275.07 (9)		
χ^2^ *(df)*	930.79 (2)			178.41 (2)		
*p*	.000			.000		

**Table 8 tbl8:** Multilevel Analysis of Coping Strategies on Stress

	Model 2			Model 3		
	(coping)			(coping + time)		
	*B*	*SE*	*p*	*B*	*SE*	*p*
Intercept	3.217	.032	.000	3.214	.031	.000
Time	−.165	.015	.000	−.165	.015	.000
Stress lag	.441	.013	.000	.443	.013	.000
Coping						
Approach	−.073	.017	.000	−.108	.042	.010
Avoidance	.145	.016	.000	.227	.039	.000
Acceptance	−.047	.014	.001	−.140	.036	.000
Close support	−.024	.010	.010	−.097	.024	.000
Distant support	.017	.008	.043	.076	.021	.000
Substance abuse	.013	.021	.553	.124	.052	.018
Self-blame	.091	.010	.000	.180	.024	.000
Interactions						
Time × Approach				.018	.020	.363
Time × Avoidance				−.043	.019	.021
Time × Acceptance				.048	.017	.005
Time × Close support				.038	.011	.001
Time × Distant support				−.031	.010	.002
Time × Substance abuse				−.061	.026	.016
Time × Self-blame				−.046	.011	.000
Residual variance						
σ_*e*_^2^ (L1: time)	.729	.015	.000	.714	.015	.000
σ_*u*_^2^ (L2: individual)	.000	.000	—	.000	.000	—
Model statistics						
*Deviance (df)*	12,036.70 (12)			11,940.32 (19)		
χ^2^ *(df)*	313.39 (7)			96.38 (7)		
*p*	.000			.000		

**Table 9 tbl9:** Four Multinomial Logistic Regression Models Exploring the Likelihood of Belonging to One of Five Stress Trajectories According to Levels of Personality, Interpersonal Skills, Cultural Adaptation, and Coping Strategies, Controlling for Baseline Stress

	Improvement in model fit		Rank order of stress trajectory likelihood				
	χ^2^(4)	*p*	1	2	3	4	5
Personality model							
Baseline stress	349.98	<.001					
Honesty-humility	6.60	.16					
Emotionality	14.80	.005	JC^2345^	MS^14^	UC^1^	MR^12^	RS^1^
Extraversion	41.09	<.001	RS^2345^	MR^1345^	MS^125^	UC^12^	JC^123^
Agreeableness	7.38	.12					
Conscientiousness	3.06	.55					
Openness	.89	.93					
Interpersonal reactivity model							
Baseline stress	336.61	<.001					
Perspective taking	8.42	.08					
Empathetic concern	17.17	.002	RS^235^	MR^1^	MS^15^	JC^5^	UC^134^
Cultural adaptation model							
Baseline stress	645.08	<.001					
Sociocultural adaptation	29.39	<.001	RS^345^	MR^345^	MS^1^	UC^12^	JC^123^
Psychological adaptation	251.00	<.001	RS^2345^	MR^1345^	MS^1245^	UC^1235^	JC^1234^
Coping strategies							
Baseline stress	643.67	<.001					
Approach coping	13.52	<.01	RS^345^	JC^5^	MR^15^	MS^1^	UC^123^
Avoidance coping	74.54	<.001	JC^345^	UC^45^	MS^145^	MR^1235^	RS^1234^
Acceptance	39.76	<.001	RS^2345^	MR^1345^	MS^125^	UC^12^	JC^123^
Close support	26.18	<.001	RS^345^	MR^345^	MS^125^	UC^12^	JC^123^
Distant support	50.30	<.001	JC^345^	UC^345^	MS^1245^	MR^123^	RS^123^
Substance abuse	9.36	.05					
Self-blame	86.11	<.001	UC^345^	JC^45^	MS^145^	MR^1235^	RS^1234^
*Note*. JC = reverse J-curve; UC = inverse U-curve; MS = mild stress; MR = minor relief; RS = resilience. Comparisons between classes were only examined for variables that significantly improve model fit (at *p* < .01). Superscripts denote significant differences in likelihood (according to Wald χ^2^ at *p* < .05).							

## Footnotes

1 AFS (originally the American Field Service) began as a service of volunteer ambulance drivers in 1914 but has since evolved into an international youth exchange organization.

2 Principal axis factoring and direct Oblimin rotation was used for both the first and second-order factor analysis.

3 An important characteristic of LCGA is that the variance of the growth parameters (i.e., intercepts and slopes) within each class is fixed to 0; they are considered to be homogeneous (Jung & Wickrama, 2008).

4 The mean of t1 and t2 stress was taken as the baseline as it was deemed to be a more stable value than stress at either one of the two time points alone. However, conducting the analysis with a single time point baseline produced the same pattern of results.

5 Comparatively smaller values for BIC and aBIC and values closer to 1 for entropy and class fit probabilities, are indicative of better fitting models. The Lo, Mendell, and Rubin (2001) likelihood ratio test (LMR-LRT) and bootstrap likelihood ratio test (BLRT) were also used to identify whether the fit of each model was a statistically significant improvement from the equivalent model with one less class (*k* – 1).

6 Many of the explanatory variables were specified as time invariant. That is, we examine how these variables (i.e., personality) as captured at a specific point in time (i.e., pretravel) covary with stress. However, we do not make any assumptions about their temporal stability.

7 Across the different analyses, the number of timewaves (Level 1) ranged from 6,297 to 6,770 and the number of individuals (Level 2) ranged from 1,788 to 2,015. The variation in sample size was due to missing timewaves and attrition.

8 Analyses in this section control for lagged stress to model change in stress. However, analyses in which lagged stress was substituted by stress baseline (stress at t1) yielded similar results.

9 Due to differences in sample size between variables, two different basic models were constructed. Variables regarding sex, nationality, and previous sojourn experience were analyzed in a model with 6,770 units at Level 1 and 2,015 units at Level 2. Variables regarding language experience, residence status, previous travel experience, and cultural experience of the family were analyzed in a model with 6,297 units at Level 1 and 1,845 units at Level 2.

10 For controls, compared with the basic model (Model 1, *deviance* = 3,285.11, *df* = 5, *N*_*L1*_ = 1,295, *N*_*L2*_ = 424) the addition of personality improved the fit (Model 2, χ^2^(6) = 62.43, *p* < .001). Associations with stress were found for emotionality (*B* = .07, *p* < .01), extraversion (*B* = −.14, *p* < .001), agreeableness (*B* = −.08, *p* < .01), and conscientiousness (*B* = −.14, *p* < .001). Cross-level interactions did not improve the model (Model 3, χ^2^(6) = 2.47, *ns*).

11 For controls, compared with the basic model (Model 1, *deviance* = 3,285.11, *df* = 5, *N*_*L1*_ = 1,295, *N*_*L2*_ = 424) the fit was not improved by the interpersonal reactivity variables (Model 2, χ^2^(2) = 2.24, *ns.*), nor the cross-level interactions (Model 3, χ^2^(2) = 1.43, *ns*).

12 For controls, compared with the basic model (Model 1, *deviance* = 3,088.89, *df* = 5, *N*_*L1*_ = 1,229, *N*_*L2*_ = 364) the addition of the coping strategies improved the model fit (Model 2, χ^2^(6) = 129.73, *p* < .001). Associations with stress were found for the approach (*B* = −.13, *p* < .001), avoidance (*B* = .12, *p* < .001), acceptance (*B* = −.09, *p* < .001), substance abuse (*B* = .06, *p* < .01), and self-blame (*B* = .10, *p* < .001) strategies, but not for social support (*B* = −.01, *ns*.). Cross-level interactions did not improve the fit (Model 3, χ^2^(6) = 4.30, *ns*).

13 A Poisson distribution or exponential distribution is defined as a discrete probability distribution that expresses the probability of a given number of events occurring in a fixed interval of time and/or space (Haight, 1967).

14 Although early return is a relatively infrequent event in our population, logistic regression is well-suited to analyze this data. Monte Carlo simulations have shown that logistic regressions can run with a minimum of 10 events per predictor variable (Peduzzi, Concato, Kemper, Holford, & Feinstein, 1996).
